# Assessment of routine surveillance data as a tool to investigate measles outbreaks in Mozambique

**DOI:** 10.1186/1471-2334-6-29

**Published:** 2006-02-21

**Authors:** Jagrati V Jani, Ilesh V Jani, Carolina Araújo, Sundeep Sahay, Jorge Barreto, Gunnar Bjune

**Affiliations:** 1 Department of Immunology, Instituto Nacional de Saúde, Maputo, Mozambique; 2 Department of General Practice and Community Medicine, University of Oslo, Norway; 3 Department of Gynaecology, Central Hospital, Maputo; 4 Department of Informatics, University of Oslo, Oslo, Norway

## Abstract

**Background:**

Measles remains a major public health problem in Mozambique despite significant efforts to control the disease. Currently, health authorities base their outbreak control on data from the routine surveillance system while vaccine coverage and efficacy are calculated based on mathematical projections of the target population. The aim of this work was to assess the quality of the measles reporting system during two outbreaks that occurred in Maputo City (1998) and in Manica Province (2002).

**Methods:**

Retrospectively, we collected data from the routine surveillance system, i.e. register books at health facilities and weekly provincial and national epidemiological reports. To test whether the provinces registered an outbreak, the distribution of measles cases was compared to an endemic level established based on cases reported in previous years.

**Results:**

There was a significant under-notification of measles cases from the health facilities to the province and national level. Register books, the primary sources of information for the measles surveillance system, were found to be incomplete for two main variables: "age" and "vaccination status".

**Conclusion:**

The Mozambican surveillance system is based on poor quality records, receives the notification of only a fraction of the total number of measles in the country and may result in failures do detect epidemics. The measles reporting system does not provide the data needed by Expanded Program on Immunisation managers to make evidence-based decisions, nor does it allow in-depth analysis to monitor measles epidemiology in the country. The progress of Mozambique to the next stage of measles elimination will require an improvement of the routine surveillance system and a stronger Health Information System.

## Background

Immunisations are among the most successful and cost effective disease prevention interventions available [1]. The great challenge of the Expanded Program on Immunisation (EPI) is to reach and to keep high levels of vaccine coverage, which contributes first to disease control and later to disease elimination and eradication.

In Mozambique, national vaccination campaigns were introduced in 1976 and the EPI from 1980. The currently targeted diseases of the Mozambican EPI are tuberculosis, poliomyelitis, diphtheria, tetanus, whooping cough, hepatitis B and measles. Measles is controlled with the administration of a single dose of the standard Schwarz vaccine at 9 months of age. Measles remains the most frequent cause of vaccine-preventable childhood death in the country.

Currently, EPI routinely offers vaccination through fixed stations and mobile teams. Since 1997, poliomyelitis mass vaccination campaigns targeted at children less than 5 years of age take place every two years in Mozambique. In the 1997 and 1999 campaigns, the measles vaccine was also given [2]. For the first time in 2005, a measles catch-up campaign that covered children from 9 months to 14 years of age was organised. This opportunity was also utilised to increase vitamin A and polio vaccination coverage in the country.

The success of EPI in Mozambique has, however, been limited by several factors that are both of extrinsic and intrinsic nature in relation to the programme. Infectious diseases control depends on broad extrinsic factors such as socio-economical stability, and accessibility and acceptability by the populations of the health services [3]. Difficulties that are intrinsic to the EPI include, among others, problems in monitoring the cold chain, the lack of quality assurance in the information and surveillance system, and the inaccuracy of demographic data used by the program to calculate vaccination targets and vaccine coverage [2]. In addition, lack of human resources, equipment and the laboratory capacity for diagnosis delay responses to epidemics.

According to the World Health Organisation [4], the measles surveillance system should be an "integrated effort to collect, process, report and use health information and knowledge to influence policy making, programme action and research"[4]. A surveillance information system should, thus, not be a static entity but a process whereby health-related data is gathered, shared, analysed, and used for decision-making in health. The ultimate goal is to use the information for designing effective disease control interventions. Two important variables that measles surveillance system needs to monitor are the "*age*" and the "*vaccination status*" of the affected individuals. Availability of such information has enabled the redefinition of vaccination policy in many countries. For instance, information about high incidence of measles in older children has led countries to introduce extra doses of vaccine for children entering school [5,6]. A lower case fatality rate can be seen in West Africa where, due to the high vaccine coverage, measles mostly occurs in vaccinated children [7].

Importantly, when measles in infants younger than nine months is frequent, a question that needs to be investigated is whether the vaccine is being administrated at the right age. Pre-immunisation measles is associated with a larger window of susceptibility in children born from mothers with a lower titre of anti-measles antibodies. This situation is expected to become increasingly common in countries like Mozambique that have high vaccine coverage, and where a significant number of women in child bearing age have vaccine-induced immunity.

At a time when Mozambique and other sub-Saharan countries are preparing to progress to the next stage of measles control, and hopefully elimination, the disease surveillance system should be prepared to play a central role in the EPI. The aim of the present study was to assess the measles reporting system during two measles outbreaks in Mozambique in 1998 and 2002.

## Methods

### Measles surveillance system in Mozambique

In Mozambique, measles diagnosis is based on the measles case definition published in the surveillance manual distributed to all health workers: "high fever (39–40°C) or a history of high fever in the preceding days, rash, inflamed eyes, and/or cough, and/or running nose". There is no case-based investigation or laboratory confirmation of cases [2].

To monitor measles in the country, the surveillance system is based on Weekly Information Bulletin System (BES). BES starts at the health-facility (HF) and flows to district and province level in paper form using pre-defined formats. From the provincial to the national level, BES reports are sent in electronic form.

The register books are used to record data from all patients seeking help at the HF and are kept there to be used as a source of information for the authorities.

The register books are formatted to contain information such as the name of the patient, age groups (0–4 years, 5–14 years, 15 years old and more), residence, symptoms and treatments prescribed. The Ministry of Health in Mozambique (MoH) recommends that for all measles cases under five years, the age and anti-measles vaccination status should be recorded in the book. For cases younger than two years, the age should be recorded in months. The recording of these data is compulsory. This recommendation is printed on every page of all register books as a footnote.

The data is registered daily in the book and at the end of the day summarised by the clinician on a data collection form that has the same format as the BES. These daily sheets are collected by a member of the preventive medicine staff at the end of each working week, and are utilised to generate the HF BES. The daily sheets are not filed and stored. The information at the district level is manually compiled on sheets of paper and sent to the province and then national levels.

The data from the HF is weekly summarised in the BES according to the format: number of measles cases and deaths under 9 months of age, number of vaccinated and non-vaccinated cases between 9 and 23 months of age, and number of cases older than 24 months of age without the vaccine status of the individuals being mentioned. The BES information does not make a distinction between inpatients and outpatients. The detailed information on the patient is only possible to find in the register book at the HF.

### Study sites and flow of information

In 1998, Maputo City, the capital of Mozambique, had 996,837 inhabitants [8]. Maputo City is, for all purposes, is considered to be a province. The health services are organised in three districts. Each district is served by health centres and one general hospital. Maputo city has one central hospital and four main private clinics.

Information flows from the health centres via the general hospitals to Maputo Health Directorate and up to the MoH. The Central Hospital and private clinics report information directly to the Maputo Health Directorate.

**Figure 1:**
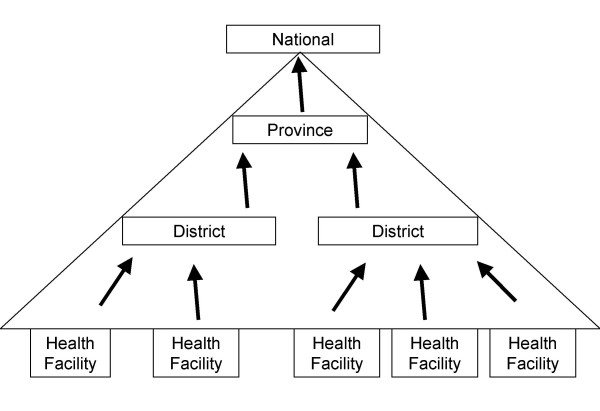
**The flow of disease surveillance information in the health system in Mozambique**. Register books at health facilities serve as the primary epidemiological data source. Data from these is collected and sent in a weekly epidemiological bulletin (BES) format to the district health authorities. Districts compile data from all health facilities and report to provinces using a district BES. Provinces compile data from all its districts and report to the national authorities using a province BES. For the sake of simplicity, only one province is shown. The national authorities collect data from all provinces and produce a national BES.

The other site, Manica Province, had 1,200,000 inhabitants in 2002 [8]. It is located in the central region and has nine districts. The health data flows from the HF to the district where the data is compiled and sent to the province and to the national level (see Figure 1).

The choice of Maputo City and two districts in the Manica Province for this study allowed for the assessment of the surveillance system in an urban and two rural settings. In Maputo City, the data was collected in all health units. The measles vaccination coverage in Maputo City in 1998 was reported at 85%. In Manica Province, two of the nine districts, Gondola and Manica were randomly chosen to be included in the study, which had measles vaccine coverage in 2002 of 63% and 93%, respectively.

### Data collection

The data was collected from two sources: the register books at the HF, and the BES reports at the province and national levels.

The HF registers were reviewed for the period between 01/01/1998 and 31/12/1998 in Maputo City, and for the period between 01/01/2002 and 31/12/2002 in Manica Province. The review was carried out retrospectively in the first quarter of 1999 and 2003 for Maputo City and Manica Province, respectively. The BES data was reviewed for the same periods at both study sites corresponding to the measles outbreaks. The professionals registering or reporting the data did not know that the system was to be monitored.

The review of measles cases and deaths in the register books at the HF covered all services including emergency services, out-patient consultation, and in-patient admissions in three districts of Maputo City and two districts of Manica Province.

### Data analysis

The data analysis included three assessments: measles case records at the HF level, reported measles cases through BES, and the epidemic alarm at the provincial level. To assess the quality of recording of measles cases in the books, two variables were checked: the age and vaccine status of the affected individuals. To assess the number of measles cases reports through BES, the reports were compared with the number of cases recorded in the register books at the HF level. To correct for double recorded cases, all cases were entered in an MS Excel data base and manually screened for double entries.

To test whether the provinces registered a measles outbreak, the temporal distribution of the reported measles cases in 1998 and 2002 were compared to an endemic level [9]. Epidemics or disease outbreaks were defined as an excess of cases of measles in a particular population, period and place [9]. To determine what was normal or to be expected, the endemic levels or ranges were established based on measles cases reported in the previous years. The Excel software program was used to develop the endemic ranges by calculation of the geometric mean (Xg) of historical rates and their confidence intervals (CI). The geometric mean was used to avoid the bias inherent in comparing measles cases during an inter-epidemic period with a low incidence, with the high number of cases during an epidemic period. The geometric mean was calculated using the measles incidence reported through the BES. The 95% CI of the geometric mean determines the upper/lower value of the endemic distribution. The CI was calculated using the following formula:

IC 95% = Xg ± t × SD÷ √n

CI95% – 95% confidence interval.

Xg – Geometric mean

t – t distribution

SD – Standard Deviation

√n – Square root of number of years minus one.

The six years (1992–1997) surveillance data made up the endemic distribution in Maputo City and ten years (1991–2001) surveillance data were used to construct the endemic distribution in Manica Province.

The attack rate was utilised to compare cases among different sites and BES target groups. The attack rate was expressed as cases per 10.000 inhabitants and was calculated dividing the number of reported measles cases by the target population of children (under 9 months, vaccinated 9–23, non vaccinated 9–23 and 24 months and older).

## Results

### Reported measles cases and deaths

According to the 1998 BES, Maputo City registered in all districts, including the Central Hospital, a total of 360 measles cases that resulted in one death. When data in the HF register books was inspected, a total of 1981 measles cases were found in Maputo City during the same year (Table 1). Of these, 87 died with a diagnosis of measles (case fatality rate of 4.4%, CI95% 3.5–5.4); 24 (27.6%) of the fatal cases occurred in children under 9 months of age. In 2002, the Manica and Gondola districts in Manica Province reported a total of 121 and 135 measles cases, respectively (Table 1). The register books at the HF level showed for the same year 161 and 180 measles cases, respectively. No death due to measles was recorded in Manica and Gondola districts of Manica Province in 2002 (Table 2).

**Table 1:** Relationship between information system and source of the information system in Maputo City and in 2 districts of Manica Province

District	Reported Cases	Recorded Cases	AR Reported (CI 95%)	AR Recorded (CI 95%)	Notification (%)
Maputo (HF = 24) Pop = 996.837 inhabit	360	1981	3.60 (+/-0.13)	19.87 (+/-0.40)	18
Manica (HF = 17) Pop = 164.000 inhabit	121	161	7.3 (+/-1.55)	9.8 (+/-1.67)	75
Gondola (HF = 9) Pop = 209.000 inhabit	135	180	6.4 (+/-1.21)	8.6 (+/- 1.40)	75

AR: Attack Rate = Number of measles cases reported/Target population × 10.000 % of Notification = Recorded cases/ Reported cases

**Table 2:** Measles cases by age, vaccination status and complications as recorded in register books at Health Facilities in Maputo City and Manica Province

Characteristics	Maputo City N (%)	Manica District N (%)	Gondola District N (%)
Average age (years)	7 (SD6)	12 (SD86)	5 (SD63)
*Age group*			
0–4 Years	870 (47)	56 (35)	90 (56)
5–14 Years	718 (39)	82 (52)	50 (31)
≥ 15 Years	268 (14)	21 (13)	20 (13)
missing	125	2	20
*Vaccine status*			
Yes	118 (6)	27 (15)	28 (16)
No	275 (14)	19 (11)	18 (10)
Without information	1588 (80)	134 (74)	134 (74)
*Clinical complications*			
Yes	215 (11)	155 (96)	95 (53)
Not recorded	1766 (89)	6 (4)	85 (47)
*Lethality*			
Deaths within health facilities	87	0	0
Total	1981	161	180

No discrepancy in the number of cases reported from the province to national level was observed for either province.

### Assessment of register books at the HF

#### Age

A total of 1981 measles cases were found in the registry books in Maputo City. Of the 1448 (73%) cases that had their age recorded in months, 713 (49%) were under two years of age.

In the district of Manica in Manica Province, 161 measles cases were recorded in the books. Of these, 55 (34%) had the age recorded in months and among these only 9 (16%) were under two years of age. In the district of Gondola in Manica Province, 180 measles cases were recorded in the books. Of the 123 (68%) that had the age recorded in months, 76 (62%) were under two years of age (See Table 2).

#### Vaccination status

Of the 870 patients less than five years old registered in Maputo City, 316 (36.3%) had information recorded about their vaccination status, and only 64 (20.3%) of these had been immunised against measles.

Manica District registered 56 patients less than five years old. Among these, 23 (41.1%) had information about vaccination status, but only 5 (21.7%) had been immunised against measles. In Gondola District, 90 measles cases occurred in children less than five years old. Of these, 26 (28.9%) had information about their vaccination status and 13 (50%) had been immunised against measles (Table 2).

### Assessment of BES measles notification system

In the BES reports, measles cases were well defined according to age groups, namely, younger than 9 months, 9–23 months (subdivided in groups of vaccinated and non vaccinated) and 24 months of age and older. The case distribution at study sites showed high measles attack rates in children 9–23 months of age (target group for measles vaccination) who had not been vaccinated against measles, followed by in children younger than 9 months old (pre-vaccination age) (see Table 3).

**Table 3:** Reported measles cases and attack rate by BES target age groups

	Maputo City 1998		Manica District 2002		Gondola District 2002	
Age group (months)	N (%)	AR	N (%)	AR	N (%)	AR
< 9	80 (22)	24	14 (12)	27	15 (11)	24
9–23 Vaccinated	50 (14)	15	6 (5)	7	26 (19)	52
9–23 non-vaccinated	51 (14)	86	26 (21)	481	35 (26)	120
≥ 24	179 (50)	2	75 (62)	5	59 (44)	3
Total	360	4	121	7	135	7

Source: BES (Epidemic Weekly Bulletin).

AR: Attack Rate = Number of measles cases reported/Target population × 10.000

### Assessment of the epidemic "warning" at the province level

**Figure 2:**
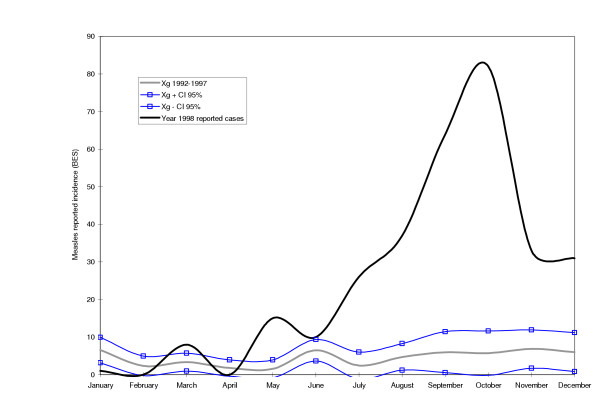
**Cases of measles during an outbreak in the year of 1998 in Maputo City**. The continuous bold line represents the reported cases of measles in 1998. The grey line represents the geometric mean of reported measles cases for the years 1992–1997. The lines with squares represent the upper and lower endemic ranges.

**Figure 3:**
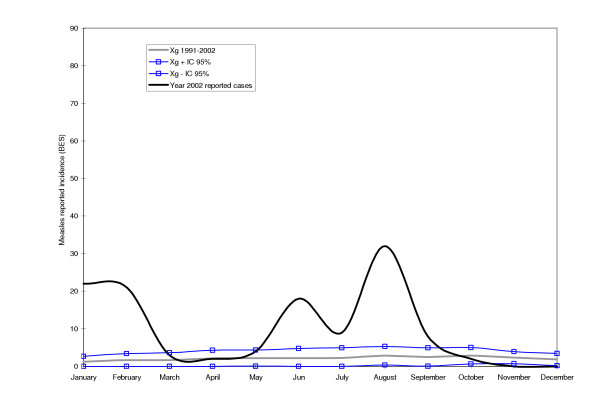
**Cases of measles during an outbreak in the year of 2002 in the Manica district of the Manica Province**. The continuous bold line represents the reported cases of measles in 2002. The grey line represents the geometric mean of reported measles cases for the years 1991–2001. The lines with squares represent the upper and lower endemic ranges.

**Figure 4:**
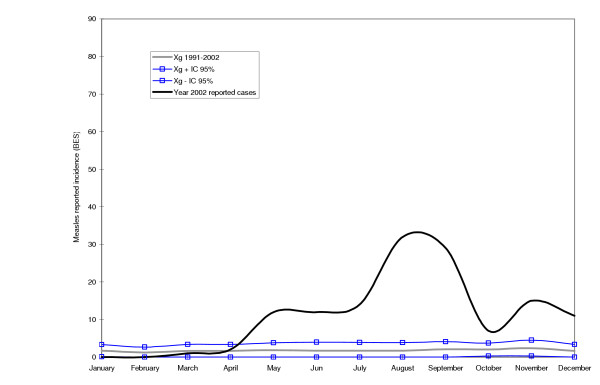
**Cases of measles during an outbreak in the year of 2002 in the Gondola district of the Manica Province**. The continuous bold line represents reported cases of measles in 2002. The grey line represents the geometric mean of reported measles cases for the years 1991–2001. The lines with squares represent the upper and lower endemic ranges.

Tested against the measles endemic distribution, both Maputo City in 1998 and the selected districts in Manica Province in 2002 were clearly undergoing an outbreak of measles (see Figures 2, 3, 4). In all cases, the peak of the outbreak occurred between the months of August and October during the dry season.

## Discussion

In 1998, the HFs in Maputo City did not report 82% of all measles patients seen in the network, while in the Manica and Gondola Districts in Manica Province there was a 25% under-notification of measles cases. The analysis of data from these two epidemics in Mozambique reveals that the BES data is unreliable, suggesting that current public health interventions are based on evidence of questionable quality. It may be argued that the findings reported in this study are not representative of the situation in all districts of the country. However, many of the factors identified to be in the root of the problems of the surveillance system, such as the format of the register books and of the BES, are common throughout Mozambique [10].

### Assessment of register books at the HF level

#### Age

The review of the register books showed incomplete age recording for children younger than two years old, and in many cases, the recording of age in months in higher age groups. Furthermore, a serious lack of age recording was observed, breaking the BES recommendations. Therefore, the average age calculated may not show the real age distribution of the affected individuals. One of the reasons that the recorded ages did not follow official recommendations is that the age group categories defined in the register books are different from those on the BES sheets.

#### Vaccination status

The lack of information in the register books on the vaccination status of patients younger than 24 months was significant. Still, the compiled information forwarded at the end of each week through BES reports showed the target age groups to be subdivided in vaccinated and non-vaccinated categories, with no column for "unknown".

Data on vaccination status in individuals 24 months of age and older is not routinely collected due to the design of currently used BES forms. While the availability of this information would allow in-depth analysis of surveillance data, one also must consider that the collection of additional data would work against the simplicity of the system. Nevertheless, the examination of available data on the vaccination status in the target group (Table 3) shows that the attack rate among non-vaccinated children is clearly high, emphasizing the need to expand the vaccination coverage in the country.

#### Notification of deaths

Based on BES information in Maputo City in 1998, the mortality was under-notified by 99% (1:87). While on one hand this under-notification of deaths is related to the under-notification of measles cases, on the other hand the register book does not provide data on how many of the recorded measles patients are hospitalised or die. In all these 87 cases found in the register books, the clinician wrote down the death events in the column that is reserved for notes on the prescribed treatment.

In 2002, Manica Province did not report any deaths due to measles. Knowing that the majority of measles cases seek help in the HF due to complications, and taking into consideration the possibility that some of the individuals were also infected by HIV [11], a high death rate could be anticipated. However, the measles cases and deaths recorded at the HF level may only represent "the ears of a hippopotamus", hiding a much bigger problem due to low coverage, about 50% of the total population, of health services in Mozambique [12].

In Manica Province measles was well recognised by mothers who typically expect that all children will get the disease once in their life. This recognition has contributed to a strong tradition for keeping children isolated in one small hut until the rash has gone. Except in case of complications, measles cases do not seek help in the HF; this situation is aggravated by long travel distances and poor means of transportation. This dilemma could explain the lack of deaths recorded in Manica Province. The tradition of isolating measles cases could also contribute to a decrease in the transmission of measles infection [13].

The high mortality recorded in Maputo City in 1998 (fatality rate 4%), may be associated with overcrowding which has been shown to increase the viral load among the secondary cases [13].

### Assessment of BES measles notification system

#### Measles cases distribution by age groups reported in BES

Two-thirds of the reported cases in the districts with epidemic outbreaks occurred in the group older than 23 months. Even though the attack rate in this group was not so high (Table 3), the high number of measles cases among them sustains the circulation of the measles virus in the community. This age group includes both vaccinated and non-vaccinated children and adults.

The remaining one third is comprised by pre-vaccination cases and those eligible for vaccination (whether vaccinated or not). The attack rate in these subgroups shows a great variability between different outbreaks and locations; for children less than 9 months of age (pre-vaccine age) it is about 25 per 10,000 inhabitants (Table 3). This is a serious problem as children younger than 9 months tend to have more severe disease, and emphasises the importance of disease prevention to decrease viral circulation among the older age groups. Children under 9 months of age account for between 11 and 22% of all measles cases reported, which is similar to the proportion observed in communities where immunisation has not been introduced [14]. However, the high attack rate in children less than 9 months would suggest that the protection provided by maternal antibodies wanes before they receive the vaccine. This assumption is compatible with the fact that a majority of children are now born to vaccinated mothers with lower titres of antibodies [15], as the EPI in Mozambique has started 24 years ago.

### Assessment of the epidemic "warning" at the provincial level

The study confirmed the existence of a measles epidemic outbreak in Maputo City in 1998 and in Manica Province in 2002. However, the epidemic curve based on BES data reflected the severe underestimation. Even with the questionable quality of BES data, it would still have been possible to release an epidemic signal in both sites. It is highly unlikely that the rise in the number of measles cases verified was due to an increase in the rate of reporting. Data for this study was collected retrospectively in the first quarter of 1999 and 2003 for Maputo City and Manica Province, respectively.

In Mozambique, laboratory confirmation of measles cases is not yet part of the surveillance system, and diagnoses are based primarily on the clinical case definition. Taking into consideration that fever and rash could lead to misdiagnosis [16-18] it is extremely important that laboratory confirmation of measles be introduced for all suspected cases in the beginning of an outbreak. This type of laboratory support for surveillance programs has been advocated by the WHO [19] and its feasibility in recent times has been significantly increased by the use of oral fluid specimens and capillary blood samples collected on filter paper [20-22]. Mozambique is now planning to introduce a case-based reporting system together with laboratory confirmation of measles. Many may consider this approach too demanding on a system that is already over-stretched. However, a similar reporting system is already in place for poliomyelitis; the addition of measles to this scheme is a challenge but is, in our opinion, a feasible task.

### Data quality assurance

The review of register books revealed serious problems of under-registration of basic information demanded by the MoH for recording and reporting of measles cases.

The information transmitted from the HF to the BES at the provincial and national levels has many deficiencies, including lack of complete records at the HF and delayed information to the district, province and national levels [23]. This situation results from the absence of a well defined working methodology and the staff being overworked at the HFs [23]. Taking into consideration that each clinician in Mozambique at the HF level sees sixty patients per day [23], and assuming that the required time per patient is twenty minutes, the clinicians need to work 20 hours per day to attend to all the patients. We believe that overwork is one of the key impediments for the improvement of the data recording system, including the process of collection and compilation. Additionally, it may also be necessary to motivate clinicians to collaborate in surveillance by communicating back the information to them and by showing them the importance of the surveillance data for directing disease control actions.

## Conclusion

Based on our study, the following key recommendations are proposed to improve the surveillance system:

- There is an urgent need for quality assurance of the current BES.

- The age groups in the register books and BES should be harmonised.

- The current format of the register books should be modified in order to accommodate all parameters required in the surveillance programs.

- The clinicians should be encouraged and motivated to fill properly the register books.

- The feasibility of gathering data on the vaccination status of children older than 24 months should be assessed.

- The health workers need to be trained in registration, collection, interpretation of data, and encouraged to take decisions based on evidence. The surveillance system should be seen not merely for gathering statistics but as providing information for action.

## List of abbreviations

EPI Expanded Program on Immunisation

BES Weekly Information Bulletin System

HF Health-facility

MoH Ministry of Health in Mozambique

CI Confidence intervals

Xg Geometric mean

## Competing interests

The author(s) declare that they have no competing interests.

## Authors' contributions

JVJ and JB conceptualized the study. JVJ and CA were responsible for data acquisition. JVJ, CA and JB were responsible for analysis and interpretation of data. IVJ provided technical input, review and project direction. JVJ, IVJ, SS and GB collaboratively wrote the manuscript. All authors read and approved the final manuscript.

## Pre-publication history

The pre-publication history for this paper can be accessed here:



## References

[B1] Lambert PH (1996). Research priorities for the WHO Global Program for Immunisation. Dev Biol Stand.

[B2] Cliff J, Simango A, Augusto O, Van Der Paal L, Biellik R (2003). Failure of targeted urban supplemental measles vaccination campaigns (1997-1999) to prevent measles epidemics in Mozambique (1998-2001). J Infect Dis.

[B3] Carr JE, Martin MR, Clements CJ, Ritchie PLJ (2000). Behavioural Factors in Immunization. Behavioural Science Learning Modules.

[B4] WHO (2000). Guidance on Needs Assessment for National Health Information Systems Development. World Health Organisation.

[B5] (1993). Measles control: a global battle. World Health Forum.

[B6] de Francisco A, Hall AJ, Unicomb L, Chakraborty J, Yunus M, Sack RB (1998). Maternal measles antibody decay in rural Bangladeshi infants--implications for vaccination schedules. Vaccine.

[B7] Samb B, Aaby P, Whittle H, Seck AM, Simondon F (1997). Decline in measles case fatality ratio after the introduction of measles immunization in rural Senegal. Am J Epidemiol.

[B8] Estatistica IN Projeccao Anual da Populacao por Distritos 1997-2010.

[B9] Bortman M (1999). [Establishing endemic levels of ranges with computer spreadsheets]. Rev Panam Salud Publica.

[B10] Chilundo B (2004). Integrating Information Systems of Disease-Specific Health Programs in Low Income Countries: The case Study of Mozambique. International Community Health.

[B11] Poulsen AG, Kvinesdal B, Aaby P, Molbak K, Frederiksen K, Dias F, Lauritzen E (1989). Prevalence of and mortality from human immunodeficiency virus type 2 in Bissau, West Africa. Lancet.

[B12] Kaarhus R, Rebelo P (2003). SWAps and Civil Society Organisations in the Health Sector in Mozambique.

[B13] Aaby P, Coovadia H (1985). Severe measles: a reappraisal of the role of nutrition, overcrowding and virus dose. Med Hypotheses.

[B14] Aaby P, Bukh J, Hoff G, Leerhoy J, Lisse IM, Mordhorst CH, Pedersen IR (1986). High measles mortality in infancy related to intensity of exposure. J Pediatr.

[B15] Parry EGRMDGG (2004). Measles. Principles of Medicine in Africa.

[B16] Nur YA, Groen J, Yusuf MA, Osterhaus AD (1999). IgM antibodies in hospitalized children with febrile illness during an inter-epidemic period of measles, in Somalia. J Clin Virol.

[B17] Cubel RC, Siqueira MM, Santos EO, Pires MF, Cruz CM, Nascimento JP (1996). Human parvovirus B19 infections among exanthematic diseases notified as measles. Rev Soc Bras Med Trop.

[B18] Lee MS, King CC, Jean JY, Kao CL, Wang CC, Ho MS, Chen CJ, Lee GC (1992). Seroepidemiology and evaluation of passive surveillance during 1988-1989 measles outbreak in Taiwan. Int J Epidemiol.

[B19] (1997). Measles eradication: recommendation from a meeting cosponsored by the World Health Organisation, the Pan American Health Organisation, and CDC.. MMWR Morb Mortal WKly Rep.

[B20] Riddell MA, Byrnes GB, Leydon JA, Kelly HA (2003). Dried venous blood samples for the detection and quantification of measles IgG using a commercial enzyme immunoassay. Bull World Health Organ.

[B21] Nigatu W, Nokes DJ, Cohen BJ, Brown DWG, Vyse AJ (2003). Pre-and post-vaccine measles antibody status in infants using serum and oral fluid testing: an evaluation of routine immunization in Addis Ababa, Ethiopia. EthiopJHealth Dev.

[B22] Nokes DJ, Enquselassie F, Vyse A, Nigatu W, Cutts FT, Brown DW (1998). An evaluation of oral-fluid collection devices for the determination of rubella antibody status in a rural Ethiopian community. Trans R Soc Trop Med Hyg.

[B23] Chilundo B, Sundby J, Aanestad M (2004). Analysing the quality of routine malaria data in Mozambique. Malar J.

